# Cloud removal in Sentinel-2 imagery using a deep residual neural network and SAR-optical data fusion

**DOI:** 10.1016/j.isprsjprs.2020.05.013

**Published:** 2020-08

**Authors:** Andrea Meraner, Patrick Ebel, Xiao Xiang Zhu, Michael Schmitt

**Affiliations:** a Signal Processing in Earth Observation, Technical University of Munich, Arcisstraße 21, 80333 Munich, Germany; b Remote Sensing Technology Institute, German Aerospace Center (DLR), Münchener Straße 20, 82234 Weßling-Oberpfaffenhofen, Germany

**Keywords:** Cloud removal, Optical imagery, SAR-optical, Data fusion, Deep learning, Residual network

## Abstract

Optical remote sensing imagery is at the core of many Earth observation activities. The regular, consistent and global-scale nature of the satellite data is exploited in many applications, such as cropland monitoring, climate change assessment, land-cover and land-use classification, and disaster assessment. However, one main problem severely affects the temporal and spatial availability of surface observations, namely cloud cover. The task of removing clouds from optical images has been subject of studies since decades. The advent of the Big Data era in satellite remote sensing opens new possibilities for tackling the problem using powerful data-driven deep learning methods.

In this paper, a deep residual neural network architecture is designed to remove clouds from multispectral Sentinel-2 imagery. SAR-optical data fusion is used to exploit the synergistic properties of the two imaging systems to guide the image reconstruction. Additionally, a novel cloud-adaptive loss is proposed to maximize the retainment of original information. The network is trained and tested on a globally sampled dataset comprising real cloudy and cloud-free images. The proposed setup allows to remove even optically thick clouds by reconstructing an optical representation of the underlying land surface structure.

## 1 Introduction

### 1.1 Motivation

While the quality and quantity of satellite observations dramatically increased in recent years, one common problem persists for remote sensing in the optical domain since the first observation until today: cloud cover. As thick clouds appear opaque in all optical frequency bands, the presence thereof completely corrupts the reflectance signal and obstructs the view of the surface underneath. This causes considerable data gaps in both the spatial and temporal domains. For applications where consistent time series are needed, e.g. agricultural monitoring, or where a certain scene must be observed at a specific time, e.g. disaster monitoring, cloud cover represents a serious hindrance.

The problem of cloud cover becomes even more apparent considering the amount of cloud coverage the Earth’s surface experiences every day. An analysis over 12 years of observations by the Moderate Resolution Imaging Spectroradiometer (MODIS) instrument aboard the satellites Terra and Aqua showed that 67% of the Earth’s surface is covered by clouds on average (King et al., 2013). Over land surfaces, the cloud fraction averages to 55%, featuring distinctive seasonal patterns. Considering the importance of these cloud occlusion percentages, it becomes clear how a successful cloud removal algorithm would greatly increase the availability of useful data. The task of detecting and removing clouds from satellite images has been tackled since the beginning of Earth observation activities, and is still today an area of active research. In this work, we present a deep learning model capable of removing clouds from Sentinel-2 images. The network design and the integration of additional Sentinel-1 SAR data makes it robust to extensive cloud coverage conditions. The model is trained on a large dataset containing scenes acquired globally, ensuring its general applicability on any land cover type.

### 1.2 Related works

The reconstruction of missing information in remote sensing data is a long-studied problem. In Shen et al. (2015), a comprehensive review of traditional techniques in provided. In the last decades, a multitude of approaches have been proposed for the specific task of cloud removal in optical imagery. Methods that follow traditional approaches can be categorized into three major clusters, namely multispectral, multitemporal and inpainting techniques. Many methods are a hybrid combination of these categories. Multispectral approaches are applied in the case of haze and thin cirrus clouds, where optical signals are not completely blocked but experience partial wavelength-dependent absorption and reflection. In such cases, surface information is partly present and can be restored, e.g. using mathematical (Xu et al., 2019, Hu et al., 2015) or physical models (Xu et al., 2016, Lv et al., 2016). Multispectral methods have the advantage of exploiting information from the original scene without requiring additional data, but are limited to filmy, semi-transparent clouds. Multitemporal approaches restore cloudy scenes by integrating information from reference images acquired with clear sky conditions (Lin et al., 2013, Li et al., 2015, Ramoino et al., 2017, Ji et al., 2018). For this, also multitemporal dictionary learning techniques can be used (Li et al., 2014). The multitemporal data may also come from different sensors on different satellites (Li et al., 2019). Multitemporal methods are the most popular as they substitute corrupted pixels with real cloud-free observations. However, problems arise when reconstructing scenes with rapidly changing surface conditions (e.g. due to phenological events) because of the time difference between the scene to be reconstructed and the reference acquisition. Inpainting approaches fill corrupted regions by exploiting surface information from clear parts of the same cloud-affected image (Meng et al., 2017). Such direct inpainting methods do not require additional images, but achieve good results only with small clouds. To mitigate this problem, the process of selecting the most suitable similar pixel to be cloned is often guided by auxiliary data, e.g. multitemporal (Cheng et al., 2014) or SAR images (Eckardt et al., 2013). Such methods deliver good results but have an increased complexity due to the requirement of multitemporal or multisensorial additional data.

In parallel to traditional approaches for cloud removal, data-driven methods using deep learning have been gaining attention recently. Many of the problems arising from traditional algorithms can be potentially solved by the end-to-end learning of deep neural networks (DNN). For example, the detection and segmentation of clouds as a preliminary step is often not required, as it can be learned implicitly by the networks. In the case of multisensor data fusion, the translation between different sensor domains can also be learned. Moreover, DNNs can be trained to cope with any type of cloud and residual atmospheric conditions. A first paper exploiting the potential of DNNs for restoring missing information in remote sensing imagery was published in Zhang et al. (2018). The method uses a spatial–temporal-spectral convolutional neural network (CNN) to restore data gaps in Landsat TM data. In the case of clouds, an additional multitemporal image of the same scene is used to support the reconstruction. Recent papers have been focusing on using a modern CNN architecture called conditional generative adversarial network (cGAN) (Mirza and Osindero, 2014). In Enomoto et al. (2017), a cGAN is trained to remove simulated clouds from Worldview-2 RGB images using NIR images as auxiliary data, while in Grohnfeldt et al. (2018) a cGAN removes simulated clouds from Sentinel-2 imagery using SAR data as additional information. An evolution of the cGAN, called Cycle-GAN, can be used to avoid the need of paired cloudy-cloudfree images for training (Singh and Komodakis, 2018). A different approach for generating cloud-free images is to perform a direct translation from SAR to optical using cGANs (Bermudez et al., 2018, Bermudez et al., 2019, He and Yokoya, 2018, Fuentes Reyes et al., 2019). Besides their powerful generative capabilities, cGANs can suffer from training and prediction instabilities when fed with bad input data (e.g. large cloud coverage), as reported in some of the referenced studies and in Mescheder et al. (2018). Based on these experiences, the work presented in this paper develops a model architecture that is robust to the presence of large and optically thick clouds in the input data. In addition to the conceptual considerations, the need of large datasets is also a prominent problem in deep learning for cloud removal. The studies cited above achieve promising results, but the used datasets are very limited and the performance is evaluated on non-independent data. An assessment of the generalization capability of the networks, i.e. their ability to remove clouds on previously unseen scenes, is therefore not directly possible. In contrast, we present and use a large dataset that is suited for a deterministic separation of images for training and testing purposes and thus provides a sound idea of how well the network will generalize to unseen Sentinel-2 data.

### 1.3 Paper structure

This paper is structured as follows. After this introductory section, the characteristics of the used dataset are presented in Section 2. The proposed methodology, including the designed neural network architecture and custom loss, are explained in Section 3. The conducted experiments and obtained results are then presented in Section 4 and further discussed in 5. Finally, a summary and conclusions are given in Section 6.

## 2 Data

While the data-driven method proposed in this paper is of generic nature and sensor-agnostic, the specific model we train and our experiments focus on satellite imagery provided by the Sentinel satellites of the European Copernicus Earth observation program (Desnos et al., 2014), as these data are globally and freely available in a user-friendly manner.

### 2.1 Sentinel-1 and Sentinel-2 missions

The cloud removal algorithm developed in this work is applied on optical data from the Copernicus Sentinel-2 mission (Drusch et al., 2012). The mission provides data for risk management, land use/land cover and environmental monitoring, as well as urban and terrestrial mapping for humanitarian and development aid. Imagery is available over all main land areas from −56° to 84° of latitude with a global revisit time of 5 days at the equator. The optical payload is called Multi Spectral Instrument (MSI) and comprises 13 spectral bands. Four 10 m high-resolution bands are placed in the visible and NIR domain for core mapping applications. Six 20 m resolution bands are used for environmental monitoring and high-level products. Three 60 m bands are used for detection and correction of atmospheric effects. The swath width is 290 km.

The SAR data used in this work originates from the Copernicus Sentinel-1 mission (Torres et al., 2012). The C-band radar instrument (5.4 GHz center frequency) on board of the two constellation satellites can operate in various modes depending on the position of the satellite and the scope of the observations. The main operational mode, called Interferometric Wide Swath (IW), is used over land surfaces and features a swath of 250 km and a resolution of 5 m in range and 20 m in azimuth direction. The combined revisit time is of 6 days. The Sentinel-1 mission was designed to provide data in all weather situations for maritime and land monitoring, emergency response, climate change and security.

### 2.2 SEN12MS-CR Dataset

The dataset presented and used in this work, called SEN12MS-CR, is an evolution of the SEN12MS dataset (Schmitt et al., 2019b). SEN12MS is publicly available and contains triplets of cloud-free Sentinel-2 optical images, Sentinel-1 SAR images and MODIS land cover maps. It was developed for common remote sensing applications, such as scene classification or semantic segmentation for land cover mapping. Using the same procedure as described in the original paper, SEN12MS-CR was created specifically as a dataset for training deep learning models for cloud removal.

SEN12MS-CR contains 169 non-overlapping regions of interest (ROIs) sampled across all inhabited continents during all meteorological seasons. The scene locations are randomly drawn from two uniform distributions, namely one over all landmasses and one over urban areas only. This introduces a bias towards urban landscapes, that are often in the focus of remote sensing studies and contain more complex patterns. The ROIs have an average size of approx. 5200×4000 px, which corresponds to 52×40 km ground coverage due to the pixels having 10 m ground sampling distance. Each ROI is composed of a triplet of orthorectified, geo-referenced cloudy and cloud-free Sentinel-2 images, as well as the correspondent Sentinel-1 image. All three images were acquired within the same meteorological season to limit surface changes. To assess the cloud coverage of the optical images, the cloud detector described in Schmitt et al. (2019a) was used. The cloud-free Sentinel-2 images have been selected with a threshold of 10% cloud coverage, while cloudy images are within 20% and 70% of cloud coverage.

The Sentinel-2 data is from the Level-1C top-of-atmosphere reflectance product and has values in the range [0, 10,000]. All 13 original bands were included. The Sentinel-1 data is from the Level-1 GRD product acquired in IW mode with two polarization channels (VV and VH). The values are $\sigma_{0}$ backscatter coefficients that have been transformed into dB scale.

**Fig. 1:**
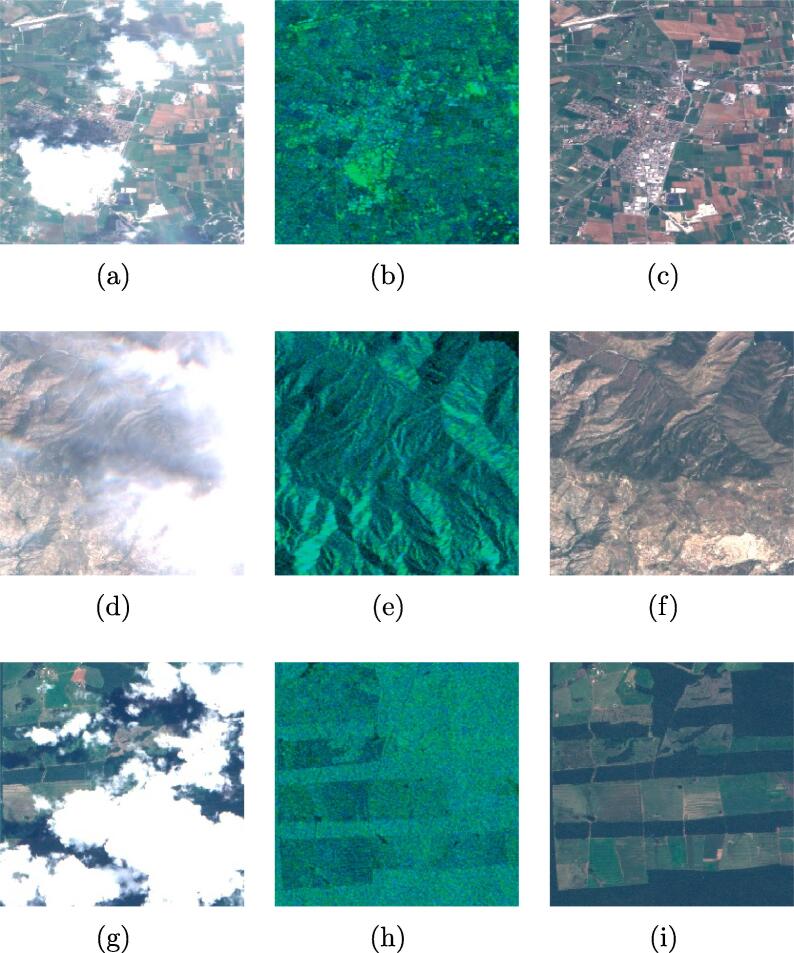
Example 256 × 256 px patch triplets from the SEN12MS-CR dataset. **(a,d,g)** are the input cloudy optical images, **(b,e,h)** are the input SAR channels, and **(c,f,i)** are the target cloud-free optical images. Throughout the paper, the shown optical images are enhanced true-color RGB composites from the Sentinel-2 10 m resolution B4-B3-B2 bands. The shown SAR images are a composite of the two polarization channels (G = VH, B = VV, R = 0).

To adapt the images for the ingestion into a CNN, the ROIs were cut into small 256×256 px patches with a 128 px stride. The amount of overlap between neighboring patches is therefore 50%. This has been chosen to maximize the number of patches extractable from an image, while still ensuring an acceptable independency. An automated and manual check of the generated patches was performed to eliminate mosaicking artifacts and other corrupted regions. The final quality-controlled SEN12MS-CR dataset contains 157,521 patches-triplets with a total of 28 layers, amounting to around 620 GB of storage size. Fig. 1 shows examples of patch triplets from the dataset. In the deep-learning based cloud removal algorithms cited in the related works, the networks are trained on datasets with clear limitations. E.g., in Enomoto et al., 2017, Grohnfeldt et al., 2018, Zhang et al. (2018) the networks are trained exclusively on simulated clouds, by using simple Perlin noise or introducing manually gaps into the imagery. In Singh and Komodakis (2018), a dataset of real unpaired cloudy and cloud-free Sentinel-2 images is used, which however is limited to the RGB channels and comprises only 20 cloudy and 13 cloud-free scenes. In Hu et al. (2015), a dataset of ten paired cloudy and cloud-free scenes acquired by Landsat-8 is used. However, the cloud-contaminated images contain only filmy, partly-transparent clouds. To the best of the authors’ knowledge, SEN12MS-CR is the first dataset used for training cloud removal networks that comprises a large and representative number of scenes sampled worldwide, with full multispectral information, containing different types of real-life clouds with their characteristic signature in all channels.

### 2.3 Train, validation and test datasets

**Fig. 2:**
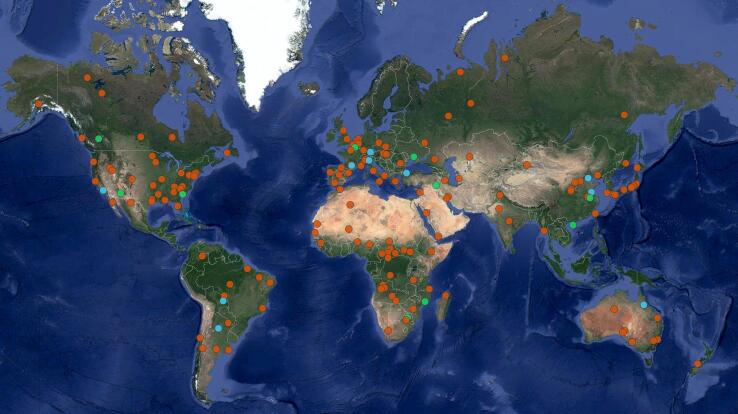
Global distribution of the 169 ROIs of the SEN12MS-CR dataset. Orange markers denote ROIs selected for training, green for validation and azure for testing. Background image credits: Google Earth/Mapmaker.

To properly assess the generalization capability of a network, a training, validation and test dataset split must be performed. For this, the 169 ROIs of SEN12MS-CR were split into 149 scenes for training, 10 for validation and 10 for testing, following a random global distribution. Fig. 2 shows the spatial distribution of the ROIs. The split according to the ROIs, rather than the patches, ensures that the three datasets are spatially and temporally completely disparate. All three datasets contain acquisitions from all meteorological seasons. A visual and automated analysis confirmed that all three datasets also have a similar distribution of cloud types and coverage amount. When separating the patches according to this split, the training dataset amounts to 134,907 patches-triplets, the validation to 11,921 and the test to 10,693.

## 3 A ResNet architecture for cloud removal

### 3.1 ResNet principle

The deep learning model used as backbone for this work is based on the popular ResNet architecture (He et al., 2016a). ResNets make use of shortcut connections, operations that skip some layers to shuttle the information to lower parts of the network, acting as a direct path for information flow. In the original ResNet case, the shortcut connection performs an additive identity mapping, i.e. the input state of a residual block is added to the output of the bypassed layers.

To further understand the residual learning rationale, let H(x) be the mapping that the skipped layers are supposed to learn as in a traditional plain network starting from the input x. By adding the additive skip connection, we let the layers explicitly learn a residual function F(x) instead:

(1) F(x)=H(x)-x.

This is helpful since it *preconditions* the task: learning a residual correction to the input has proven to be easier for current optimizers than learning the entire input–output mapping from scratch. This is especially true when the optimal mapping for a residual unit is actually close to the identity, i.e. when the network has to just reproduce the input data in the output.

### 3.2 Residual learning for cloud removal

For the task of cloud removal, the residual skip connections of a ResNet are helpful in several ways:

• Filmy clouds correction: Residual learning offers a clear advantage in the presence of filmy clouds. In this case, the network has to learn only an additive correction that compensates for the thin cloud disturbance in the overcast regions. Through the band concatenation, the network is able to access both the spectral and spatial features; the still partially present ground information acts as a good preconditioning for the restoration process.
• Cloud-free parts reproduction: Due to the large field of view and the comparably small size of clouds, satellite images are typically a mixture of cloudy and cloud-free regions. Over clear-sky regions, the residual connections offer a direct path to transfer unmodified surface information directly to the output.
• Stability of prediction: a ResNet architecture for cloud removal is robust to the presence of large and optically thick clouds in the input data. Even if an input cloudy image is mostly covered by opaque clouds, the network is at least able to reproduce adequately the cloud-free sections. C-GAN based methods (e.g. see Singh and Komodakis, 2018), tend to suffer from prediction instabilities or complete failures with bad input data.
• Optimized learning of deep models: High representational capacity given by a large number of layers and filters in CNNs is required to reconstruct the signal under thick clouds, where complex structures need to be restored. The ResNet architecture allows to optimize large and deep models in a comparably fast way and with good performance (He et al., 2016b).

### 3.3 DSen2-CR model

**Fig. 3:**
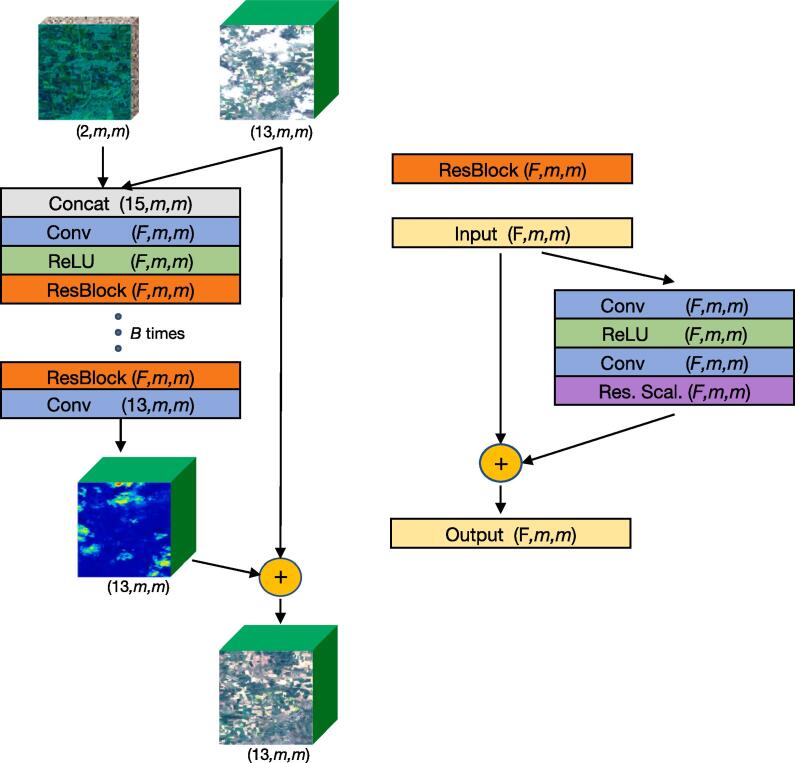
Left: DSen2-CR model diagram. Right: Residual block design. For each part of the network, the number of layers and the two spatial dimensions are indicated inside parentheses. Since the network is fully convolutional, it can accept input images of arbitrary spatial dimensions *m* during training and prediction time. *F* indicates the selected feature dimension and *B* the selected number of residual blocks included in the network.

The proposed model, called DSen2-CR, is based on the super-resolution Deep Sentinel-2 (DSen-2) ResNet presented in Lanaras et al. (2018), which is itself derived from the state-of-the-art single-image super-resolution EDSR network (Lim et al., 2017). Similarly to super-resolution, cloud removal can be seen as an image reconstruction task, where missing spatial and spectral information has to be integrated into the image to restore the complete information content. To guide the reconstruction process under thick, optically impenetrable clouds where no ground information is available, DSen2-CR leverages a SAR image as a form of prior. For this, a Sentinel-1 image of the same scene is introduced to the network as an additional input. The image's SAR channels are simply concatenated to the other channels of the input optical image. The highly non-linear SAR-to-optical translation, as well as the cloud detection and treatment, are learned and performed implicitly inside the network. The training is done in an end-to-end setup, and a cloud-free image of the same scene is presented to the network as a target for the loss computation. Fig. 3 shows a diagram of the DSen2-CR model and the used residual block design. In the following, further properties and peculiarities of the network are described:

• **Long skip connection:** An additive shortcut shuttles the input cloudy image to an addition layer right before the final output, as originally proposed in Lanaras et al. (2018). This basically means that the entire network is learning to predict a residual map that contains corrections to each pixel of the input cloudy image. In the case of a clear sky input or filmy clouds, the predicted corrections will be minor or non-existent. Conversely, for thick clouds with bright appearance, the corrections will be larger.
• **Residual blocks:** The main part of the network consists of several residual units stacked in sequence. The specific number of units *B* in the network is a hyperparameter that defines the depth of the network. The residual units each contain four layers and an addition layer for the residual connection. The four skipped layers are a 2D convolution layer with subsequent ReLU activation, a second 2D convolution layer and a final residual scaling layer (see next point). Only one ReLU activation is used after the first convolutional layer but not after the second, since the network is supposed to predict corrections that can be both positive and negative. For both convolutional layers, 3 × 3 kernels are used, following the general community trend to use smaller kernels in deeper models (Lanaras et al., 2018). The output feature dimension *F*, i.e. the number of different filters, is fixed for all units and is a hyperparameter. A stride of one pixel and zero padding is always used in order to maintain the spatial dimensions of the data throughout the network.
  Compromising between representational capacity and computational complexity, as well as considering own experiments and the reported experiences in Lanaras et al., 2018, Lim et al., 2017, B=16 residual units with F=256 features were selected as a baseline for the DSen2-CR architecture.
• **Residual scaling:** This residual scaling layer is a custom layer that multiplies its inputs with a constant scalar. First proposed in Szegedy et al. (2017), this activations scaling has the effect of stabilizing the training without introducing additional parameters, such as in batch normalization layers. The value of 0.1 is selected for the scaling constant in this work.
• **Additional convolutions:** At the beginning of the network, a concatenation layer stacks vertically the input optical and SAR layers to enable the joint processing. After this, a 3 × 3 convolution layer with ReLU activation is introduced to treat the concatenation before the data is passed through the residual blocks. After the last residual unit, a final 3 × 3 convolution restores the spectral dimensions to match the number of bands of the optical image before reaching the residuals addition layer.

Several experiments on the network structure and residual block design confirmed the validity and quality of the original DSen-2 architecture. The modifications in DSen2-CR with respect to the original network include the adaptations required to accommodate the two SAR input layers used for guiding the reconstruction, the different number of input and output optical channels, and network depth as described above.

### 3.4 Cloud-adaptive regularized loss

**Fig. 4:**
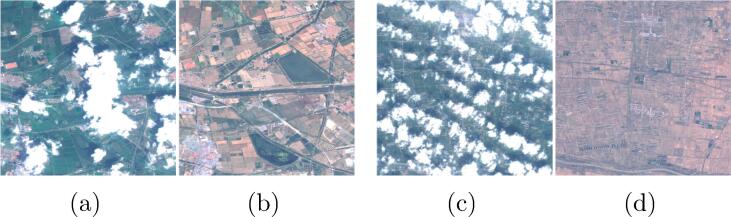
Example images showing changes in surface conditions between the input cloudy acquisitions **(a,c)** and the target cloud-free images **(b,d)** taken on a different date.

As described in the dataset section, the input cloudy image and the target cloud-free optical images have been acquired on different days, but within the same meteorological season. Although the time difference is limited, changes in the surface conditions between the images can still often be observed, especially on agricultural landscapes (see Fig. 4). Since the objective of a cloud removal algorithm is to restore ground information below clouds without modifying clear parts, it is of strong importance that the most possible information from the input image is retained in the output. To minimize the influence of ground changes in the target image, a custom training loss was developed in this work.

Following the recommendation of Lanaras et al. (2018), the $L_{1}$ metric (mean absolute error) was used as a basic error function due to the robustness to large deviations and the high dynamic range of the Sentinel-2 data. Defining the predicted output image as P and the cloud-free target image as T, the classic target loss $L_{T}$ based on the simple $L_{1}$ distance between prediction and target can be formulated as

(2) $$L_{T}=\frac{∥P-T{∥}_{1}}{N_{\mathrm{tot}}},$$

with $N_{\mathrm{tot}}$ being the total number of pixels in all channels of the optical images. The optimization on this plain $L_{1}$ loss is simple and straightforward, but it has a drawback: the network is induced to learn, predict and apply unwanted surface changes, due to being trained on multi-temporal data with changing ground conditions. To reduce these artifacts, a novel loss principle was developed. The idea is to incorporate a binary cloud and cloud-shadow mask (CSM) into the loss computation, and use this information to steer the learning process towards a maximized retainment of input information. This custom loss, which we call Cloud-Adaptive Regularized Loss ($L_{\mathrm{CARL}}$), is formulated as

(3) LCARL=∥CSM⊙(P-T)+(1-CSM)⊙(P-I)∥1Ntot︷cloud-adaptive part+λ∥P-T∥1Ntot︷target reg.part

with P,T,I denoting respectively the predicted, target, and input optical images. The CSM mask has the same spatial dimensions of the images and pixel values 1 for clouds and shadows pixels or 0 for uncorrupted pixels. 1 denotes a matrix of ones with the same spatial dimensions as the images and the CSM. The multiplications marked with ⊙ between the CSM and the image differences are element-wise and applied over all channels. In the cloud-adaptive part, the mean absolute error loss is computed w.r.t. the target image for cloudy or shadowed pixels of the input image, and w.r.t. the input image itself for clear-sky pixels. With this, the network learns that it shall optimize the predictions to match the cloud-free parts of the input, and use the multitemporal information only when needed, i.e. for the cloud and shadow reconstruction. However, when training with this cloud-adaptive part only, it was observed that the network introduced artifacts in the predicted images due to a too precise learning of the mask. To avoid this effect, an additional target regularization term in the form of a classic mean absolute error loss between prediction and target (equivalent to $L_{T}$ in Eq. (2)), was added to the loss function. This additional loss induces the network to learn to produce images that still have a natural, smooth appearance similar to the target image. The regularization factor λ, that scales this target regularization term in Eq. (3), is a hyperparameter that effectively balances the input information retainment and the prediction artifacts. After extensive tuning, the value of λ=1 was found to provide the best trade-off.

The authors have found that a methodically similar context-aware loss was proposed in Li et al. (2019) in a more generic image processing context. The novelty of the described $L_{\mathrm{CARL}}$ approach still resides in how a cloud and cloud-shadow mask is created and used in the context of cloud removal, with the specific intent of guiding and improving the reconstruction performance.

**Fig. 5:**
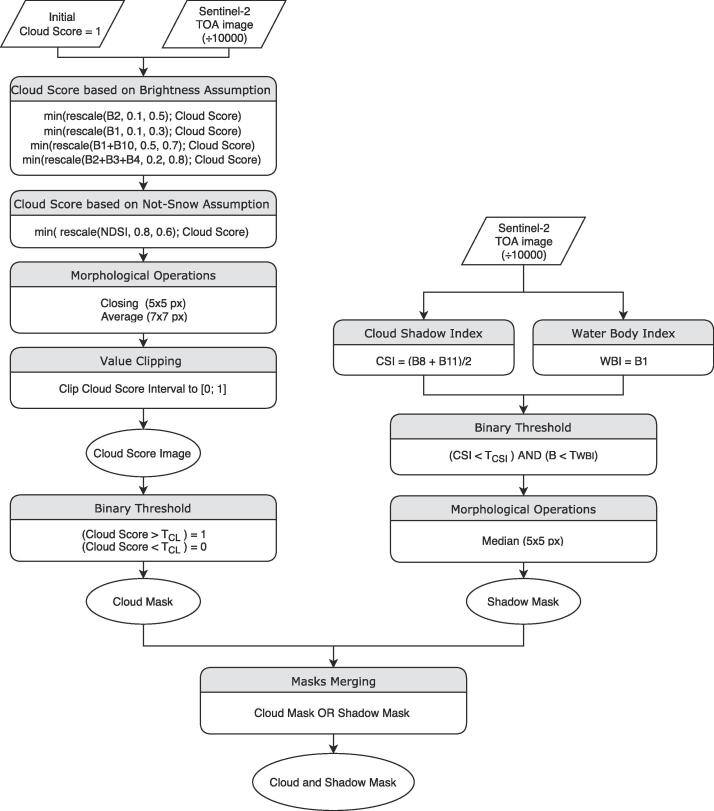
Flowchart of the cloud (left stream) and shadow (right stream) detectors employed for the mask creation used in the $L_{\mathrm{CARL}}$ loss.

For the CSM mask implementation, which is needed during training, a combination of the methods proposed in Schmitt et al. (2019a) (cloud detection) and in Zhai et al. (2018) (cloud-shadow detection) was used. Fig. 5 shows the flowchart of the different processing steps for the mask creation. The threshold $T_{\mathrm{CL}}=0.2$ for the cloud binarization was selected after a visual evaluation. The thresholds for the cloud detection were computed using the parameters $T_{\mathrm{CSI}}=\frac{3}{4}$ and $T_{\mathrm{WBI}}=\frac{5}{6}$. The threshold values were chosen in a conservative manner to reduce false negative detections. We refer to the original papers for further details on the algorithm implementations.

### 3.5 Preprocessing and training setup

Prior to the ingestion into the network, the images are value-clipped to eliminate small amounts of anomalous pixels. The clipping range for the Sentinel-2 bands is [0, 10,000], for the Sentinel-1 VV and VH polarizations it is [−25,0] and [−32.5,0], respectively. For the Sentinel-2 data, a division by 2000 is further applied to all bands to ensure numerical stability (Lanaras et al., 2018). Similarly, the Sentinel-1 values are shifted into the positive domain and scaled to the range [0, 2] to approximately match the optical data values distribution after scaling. As a data augmentation step, random rotations and flips are applied to the images before the ingestion.

The training framework has been implemented in the Keras open source deep-learning Python library with Tensorflow (Abadi et al., 2016) as backend, basing on the code from (Lanaras et al., 2018). The models were trained on a NVIDIA DGX-1 machine containing 8 P100 GPUs.

The weights of the network have been initialized using a uniform He distribution (He et al., 2015), and the biases were initialized to zero. Several tests with common optimizers showed that the Adam algorithm with integrated Nesterov momentum (Dozat, 2015) delivers the best performance. After a systematic search, the optimal learning rate has been found to be $7\cdot 10^{-5}$ for a batch size of 16.

## 4 Experiments & results

For a quantitative evaluation, we report the error metrics obtained by evaluating the results from the entire hold-out test dataset on different network configurations in the following. The used metrics are the mean absolute error (MAE) and the root-mean-square error (RMSE) in units of top-of-atmosphere reflectance $\rho_{\mathrm{TOA}}$, the peak signal-to-noise ratio (PSNR) in decibel units, the spectral angle mapper (SAM) (Kruse et al., 1993) in degrees, and the unitless structural similarity index (SSIM) (Wang et al., 2004). The MAE, RMSE, and PSNR are popular evaluation metrics for pixel-wise reconstruction quality. The SAM gives a measure of the spectral fidelity of the reconstructed images, while the SSIM assesses spatial structure quality based on visual perception principles.

### 4.1 Influence of SAR-optical data fusion

**Fig. 6:**
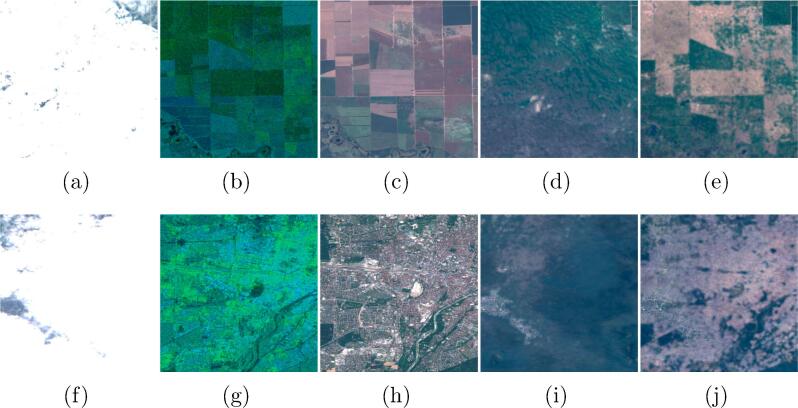
Example images showing the influence of the SAR input on an agricultural and an urban scene under heavy cloud coverage. **(a,f)** show the cloudy input images, **(b,g)** the input auxiliary SAR images, **(c,h)** the target cloud-free images. **(d,i)** are the model predictions without the SAR input, and **(e,j)** are the predictions of the full DSen2-CR model including the SAR input.

Several experiments were dedicated to verify the usefulness of the SAR-optical data fusion setup used in DSen2-CR. For this, we performed a full network training with and without including the SAR auxiliary input. In Fig. 6, example results obtained on the hold-out test dataset are visually compared. For better comparability, both networks were trained using the plain $L_{1}$ loss $L_{T}$. It can clearly be seen that the results which make use of SAR-optical data fusion contain much more structure than the results relying on pure optical-to-optical image translation.

Especially large structures that have regular shapes and a distinctive appearance in the SAR image, e.g. the large fields in the agricultural example scene, are correctly included in the predicted image. Complex objects, e.g. in cityscapes, are harder to integrate due to their more complicated patterns. Here, the model is able to reconstruct the scene only on a coarse scale. For example, the urban example area, with the core town and the river entering from the south, is at least roughly recognizable in the predicted image generated using the SAR information, whereas it is not reconstructed at all if no SAR data is used.

Considerations about the effectiveness of the SAR input can also be made by evaluating the test results reported in Table 1. Here, results from experimental training runs without SAR are provided alongside the full configurations. Comparing the numbers, the network with the SAR input scores better results for most evaluated metrics. Interestingly, however, the networks without SAR achieve lower MAE and SAM *reproduction* errors. This indicates that the network partly integrates SAR information also when reproducing cloud-free regions of the input image. Since such artifacts do not have a correspondence in the original optical image, this leads to a higher reproduction error (for MAE and SAM respectively 2% and 3% using $L_{T}$, and 9% and 2% using $L_{\mathrm{CARL}}$). However, the benefit in terms of *reconstruction* error (approx. 6% for MAE and 11% for SAM for both losses) outbalances this problem, making the SAR-optical data fusion concept beneficial for the overall cloud removal task.

**Table 1:** Quantitative results computed on the hold-out test dataset. Results are reported for the proposed DSen2-CR network in different configurations: trained on the proposed $L_{\mathrm{CARL}}$ loss, trained on the plain L1 target loss $L_{T}$, and trained on $L_{\mathrm{CARL}}$ and $L_{T}$ but without the SAR input. In the tables, *Target* refers to the error computed between the predicted image and the target cloud-free image. This is the loss as optimized using $L_{T}$. *Reprod* denotes the reproduction error, namely the error between the predicted image and the clear parts of the input image. This is part of the $L_{\mathrm{CARL}}$ loss that is explicitly optimized. *Recon* is the reconstruction error, namely the error between the predicted image and the target image inside the reconstructed clouds and shadow regions.

(a) Test results on pixel-wise metrics					
	MAE ($\rho_{\mathrm{TOA}}$)			RMSE ($\rho_{\mathrm{TOA}}$)	PSNR (dB)
Method	Target	Reprod	Recon	Target	Target
DSen2-CR on $L_{\mathrm{CARL}}$	0.0290	0.0204	**0.0266**	0.0366	28.7
DSen2-CR on $L_{T}$	**0.0270**	0.0398	**0.0266**	**0.0343**	**29.3**
DSen2-CR on $L_{\mathrm{CARL}}$ w/o SAR	0.0306	**0.0188**	0.0282	0.0387	27.6
DSen2-CR on $L_{T}$ w/o SAR	0.0284	0.0389	0.0281	0.0361	28.8

*pix2pix*	*0.0292*	*0.0210*	*0.0274*	*0.0424*	*28.2*

(b) Test results on spectral and structural fidelity metrics.				
	SAM (°)			SSIM
Method	Target	Reprod	Recon	Target
DSen2-CR on $L_{\mathrm{CARL}}$	8.15	3.94	**8.04**	0.875
DSen2-CR on $L_{T}$	**8.07**	6.33	8.13	**0.878**
DSen2-CR on $L_{\mathrm{CARL}}$ w/o SAR	8.98	**3.86**	8.97	0.870
DSen2-CR on $L_{T}$ w/o SAR	8.97	6.17	9.05	0.873

*pix2pix*	*13.68*	*13.93*	*12.67*	*0.844*

This becomes also clear by a qualitative analysis of the produced images. In Fig. 6, exemplary detail patches under thick cloud cover are presented. By comparing the predicted images with and without SAR prior, the gain in structural content provided by the SAR fusion is clear.

### 4.2 Influence of the cloud-adaptive regularized loss

**Fig. 7:**
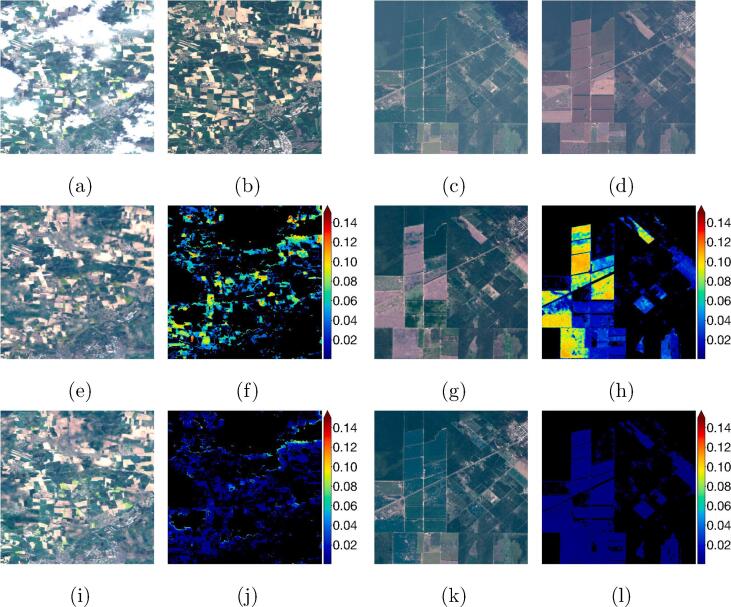
Example images showing the influence of the $L_{\mathrm{CARL}}$ loss on two agricultural scenes. **(a,c)** are the input images. **(b,d)** are the target images. **(e,g)** are the predictions obtained by training the DSen2-CR model on the plain $L_{T}$, and **(i,k)** are the predictions obtained using $L_{\mathrm{CARL}}$. **(f,h)** and **(j,l)** are the respective reproduction error maps in units of top-of-atmosphere radiance. The areas within the cloud and cloud-shadow mask (CSM) are depicted in black.

One of the main contributions of this work is the design of the so-called cloud-adaptive regularized loss $L_{\mathrm{CARL}}$. This custom loss is cloud- and shadow-aware and introduces an optimization w.r.t. to the *input* image, in order to retain the most possible amount of information from the uncorrupted input regions. To assess the effectiveness of this proposed loss, we compare the predictions of DSen2-CR models trained on $L_{\mathrm{CARL}}$ to models trained only on the plain $L_{T}$. Fig. 7 shows example images from the test dataset containing two different agricultural landscapes subject to substantial surface changes between the input and the target images. By comparing the RGB composites of the results obtained using $L_{T}$ and $L_{\mathrm{CARL}}$ with the input and the target images, it becomes clear how the network optimized on $L_{\mathrm{CARL}}$ is able to optimally retain input information and limit the artifact generation in the predicted images. In the left image series, for example, the blooming rapeseed fields captured in the input image are kept in a bright yellow color by the $L_{\mathrm{CARL}}$, while being changed to green by $L_{T}$.

The shown error maps are the pixel-wise mean absolute error between the predicted image and the cloud-free parts of the input image. In the following, we call this measure *reproduction error*, i.e. the error introduced by the network while reproducing the already cloud-free parts of the input image into the prediction. A low reproduction error indicates an optimal retainment of useful input information. Moreover, it signifies a low artifact generation caused by the training on multi-temporal images with differing ground conditions.

Observing the reproduction error maps shown in the figure, the influence of the adaptive loss is evident, with predictions from $L_{T}$ showing much higher reproduction errors in the clear-sky pixels. An evaluation of the final test results in Table 1 shows that model trained on the $L_{\mathrm{CARL}}$ loss achieves 49% less MAE reproduction error and 38% less SAM reproduction error w.r.t. to the network optimized on $L_{T}$. The reconstruction errors between the two models are comparable, showing that $L_{\mathrm{CARL}}$ does not affect negatively the cloud reconstruction performance of the network while optimizing the information retainment capabilities. Considering these observations, we conclude that the usage of $L_{\mathrm{CARL}}$ in the optimization process is beneficial for the cloud removal task. This is particularly true for agricultural areas, which exhibit phenological changes even within the limited time span lying between the acquisition of the cloud-affected image and the acquisition of the cloud-free target image. It may be noted, however, that using $L_{T}$ naturally leads to better results in target-only based metrics (here RMSE, PSNR and SSIM) since the optimization and the evaluation is performed on the same objective. This however does not necessarily signify an improvement in the overall cloud removal performance, due to the artifact generation in cloud-free part as discussed above.

### 4.3 Comparison against baseline model

**Fig. 8:**
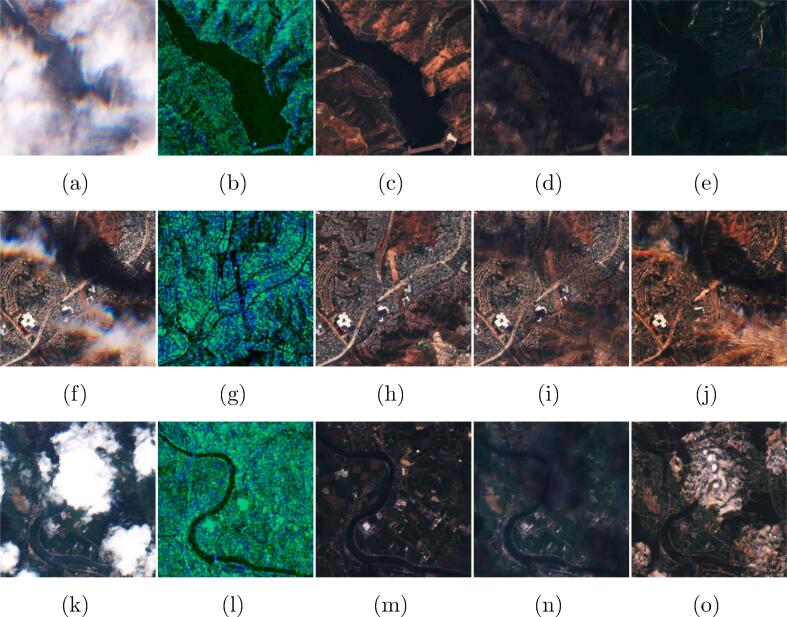
Example images comparing the cloud removal results of our model with the pix2pix baseline network, both models receiving cloudy optical and SAR data as input. **(a,f,k)** show the cloudy input images, **(b,g,l)** the input auxiliary SAR images, **(c,h,m)** the target cloud-free images. **(d,i,n)** are the predictions of our DSen2-CR model, and **(e,j,o)** are the predictions of the pix2pix baseline. The results show that our model achieves higher-fidelity results, removes cloud shadows better and is less prone to artifacts.

In order to compare our model against a standard baseline, we utilized the popular pix2pix architecture (Isola et al., 2017) that was as well adapted in previous studies on cloud removal (Grohnfeldt et al., 2018, Bermudez et al., 2018). The architecture of our baseline consists of a U-net (Ronneberger et al., 2015) generator and a PatchGAN discriminator (Karacan et al., 2016). The generator takes 13 channel multi-spectral optical and dual-polarimetric SAR patches as input, both of size 256×256 pixels. The discriminator takes as input a concatenation of dual-polarimetric SAR patches, the 13-channel multi-spectral cloudy and the real or generated cloud-free patches. SAR patches are clipped to values [−25, 0] and rescaled to range [−1, 1]. Optical patches are clipped to values [0, 10,000] and rescaled to range [−1, 1]. The network weights are initialized with a Normal initialization and biases are set to zero, The network is trained on the complete training set via ADAM (Karacan et al., 2016) (momentum 0.5) for a total of 10 epochs with the original GAN loss (Isola et al., 2017) and an $L_{1}$ loss, weighted with $\lambda_{L_{\mathrm{GAN}}},\lambda_{L_{1}}=1,100$ as in the original study (Goodfellow et al., 2014). Batch normalization (Ioffe and Szegedy, 2015) is applied to the generator. The initial ${\mathrm{Niter}}_{\mathrm{init}}=5$ epochs are trained at a learning rate of ${\mathrm{lr}}_{\mathrm{init}}=2\cdot 10^{-4}$, followed by ${\mathrm{Niter}}_{\mathrm{decay}}=5$ epochs with lambda learning rate decaying ${\mathrm{lr}}_{\mathrm{init}}$ by the multiplicative factor $\lambda_{\mathrm{decay}}=1.0-\max(0,2+\mathrm{epoch}-{\mathrm{Niter}}_{\mathrm{init}})/({\mathrm{Niter}}_{\mathrm{decay}}+1)$, where *epoch* denotes the number of the current epoch. Both the quantitative results presented in Table 1 and the example images shown in Fig. 8 illustrate the superiority of the our DSen2-CR approach – especially in terms of spectral and structural fidelity.

### 4.4 Application of the full model on large scenes

**Fig. 9:**
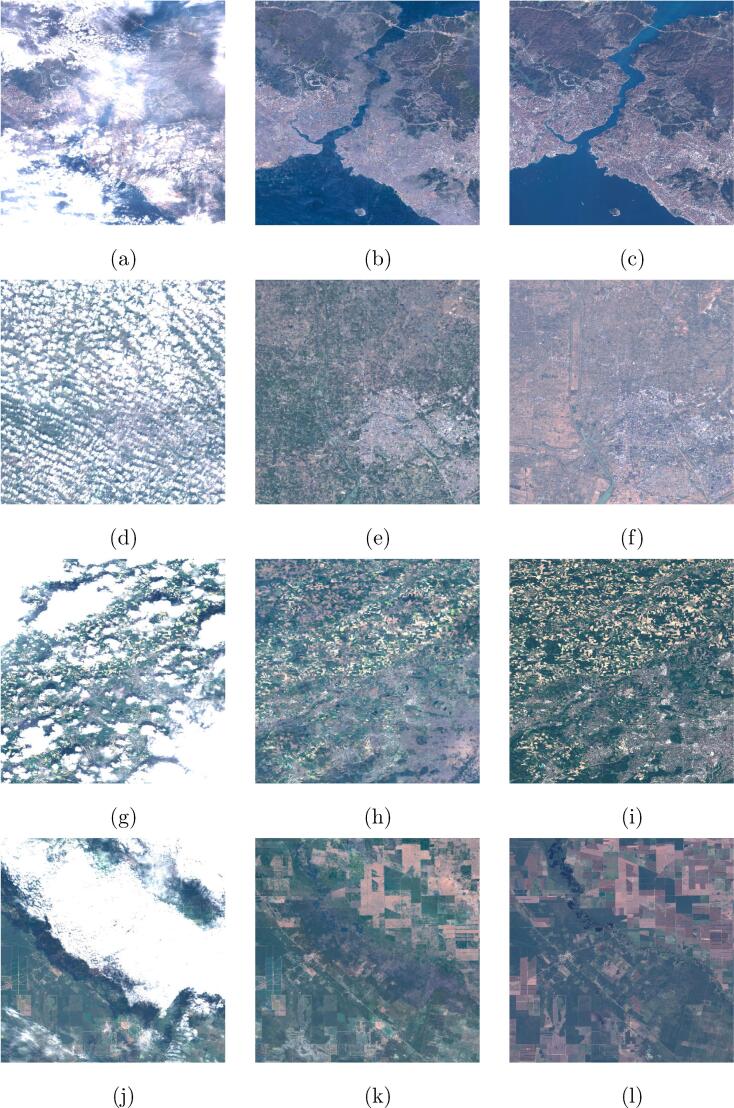
Example results from the final setup of DSen2-CR using the $L_{\mathrm{CARL}}$ loss. **(a,d,g,j)** are the input cloudy images, **(b,e,h,k)** the predicted images, and **(c,f,i,l)** the target images.

**Fig. 10:**
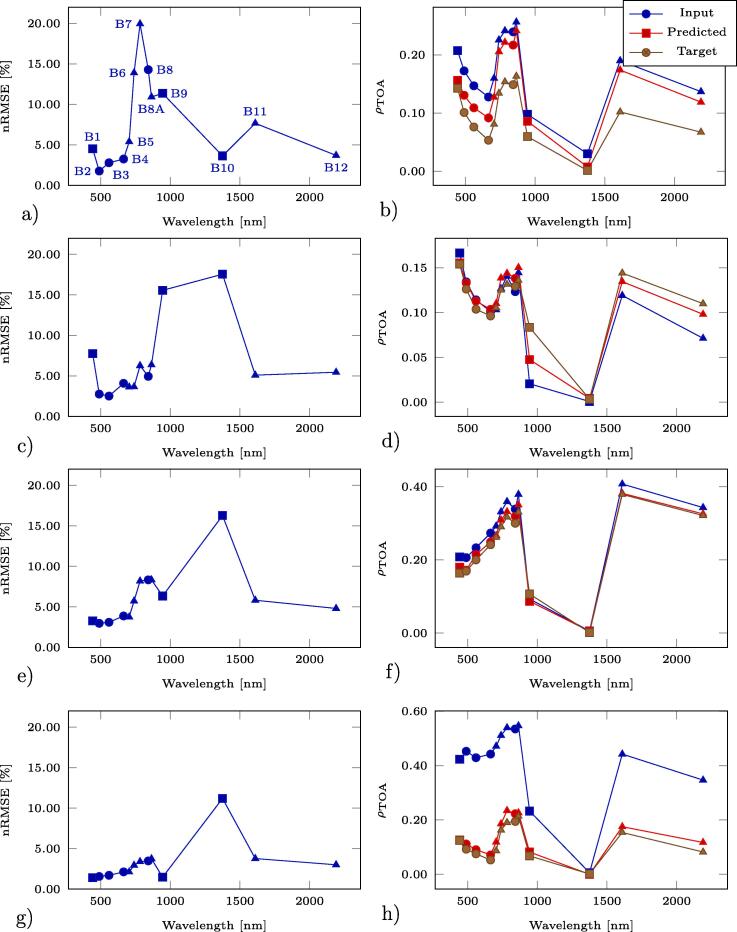
Left column: channel-wise normalized root-mean-square error (nRMSE) in units of percentage for each image shown in Fig. 9. The normalization was performed using the value range of each band. Right column: Pixel spectra of the central pixel in the respective input, predicted, and target images. The point markers denote the band resolution: circles for 10 m, triangles for 20 m, and squares for 60 m resolution. **(a)** additionally contains labels for each band following the Sentinel-2 bands na.ming convention.

**Fig. 11:**
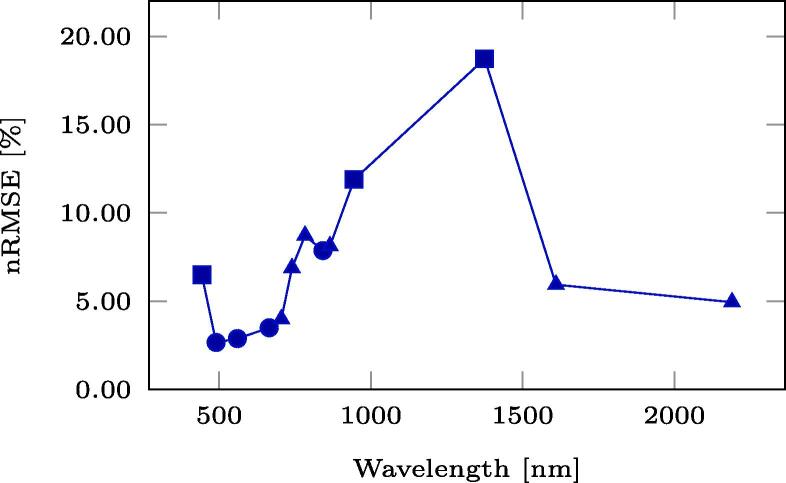
Average of channel-wise nRMSE over all test images.

**Fig. 12:**
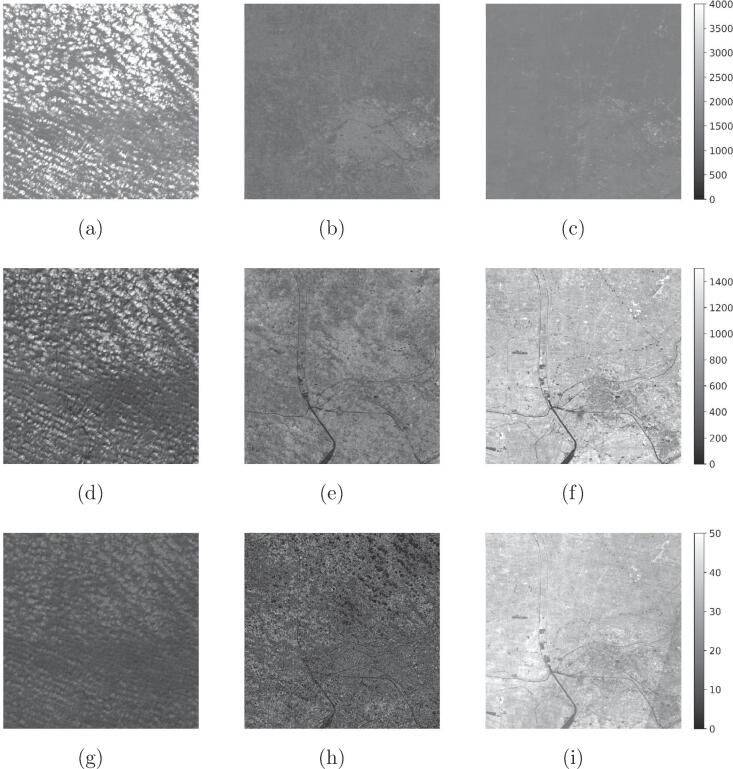
60-m resolution channels (B1, B9, B10) for the second image in Fig. 9. Left column: input image. Central column: prediction. Right column: target image.

For a qualitative evaluation of the operational performance of the full DSen2-CR model trained on the $L_{\mathrm{CARL}}$ loss including the SAR input, Fig. 9 shows a selection of large reconstructed scenes, i.e. images larger than the 256×256-pixel patches the model was trained and validated on. These scenes were concatenated from patches belonging to the hold-out test dataset. To assess the reconstruction performance in all optical channels, Fig. 10 shows the normalized root-mean-square errors (nRMSE) averaged over each optical channel for the pictures shown in Fig. 9. The normalized representation was chosen for better interpretability, since the absolute RMSE spectra have been observed to correlate with the reflectance spectra. Additionally, in Fig. 10 we also show spectra of the central pixel of each image. To assess the overall band-wise reconstruction quality, averages over all test images of each band-wise normalized RMSE are shown in Fig. 11. It can be seen that the channels, which experience the overall worst reconstruction quality, are B10, followed by B9, and B1 – all of which observe the atmosphere rather than the land surface (see Fig. 12).

Therefore, the performance of the model in reconstructing ground information even below large and thick clouds can still be appreciated on a large scale. The central pixel of the last image (Fig. 10h) is a cloudy pixel, which can be recognized by the high reflectance values of the input image. Here it can be seen how the model successfully reconstructs the entire cloud-free pixel spectrum. For the third image (Fig. 10f) the reconstructed spectrum is also very close to the target, while for the first two images (Fig. 10, Fig. 10b) the reconstruction lies between input and target, either due to prediction inaccuracy or due to the partial retainment of input information induced by the $L_{\mathrm{CARL}}$ loss.

## 5 Discussion

As the results summarized in Section 4 show, the DSen2-CR network is generally capable of removing clouds from Sentinel-2 imagery. This is not limited to a purely visual RGB representation of the declouded input image, but includes the reconstruction of the whole pixel spectrum with an average normalized RMSE between 2% and 20%, depending on the band. It should be noted, however, that the worst reconstruction results are achieved for the 60 m-bands, which are not meant to observe the surface of the Earth, but rather the atmosphere: B10, which shows the worst normalized RMSE values, is dedicated to a measurement of Cirrus clouds with a short-wave infrared wavelength; B9 is dedicated to measuring water vapor, and B1 is supposed to deliver information about coastal aerosoles (cf. Fig. 11). Since the SAR auxilary image uses a C-band signal with much longer wavelength, it is not affected by those atmospheric parameters at all and just provides information about the geometrical structure of the Earth surface. This, of course, distorts the reconstruction of the atmosphere-related Sentinel-2 bands, as can be seen in Fig. 11. However, most classical Earth observation tasks, which benefit from a cloud-removal pre-processing step, do not employ those bands anyway and restrict their analyses to the 10 m- and 20 m-bands, which provide actual measurements of the Earth surface. Thus, the inclusion of the SAR auxiliary image can definitely be deemed helpful, which is also confirmed by the numerical results listed in Table 1 and the qualitative examples shown in Fig. 6: The overall best result with respect to pure numbers is achieved when the classic loss $L_{T}$ and SAR-optical data fusion are used. The new cloud-adaptive loss $L_{\mathrm{CARL}}$, however, leads to a much better retainment of the original input and introduces less image translation artifacts, which are usually caused by training on images with a temporal offset. In summary, the combination of SAR-optical data fusion and the cloud-adaptive loss $L_{\mathrm{CARL}}$ provides the results that generalize best to different situations and also provide reliable cloud-removal for both rather thick clouds and vegetated areas which exhibit phenological changes. In the worst case, i.e. when the scene is comprised of complex patterns and the cloud cover is optically very thick, the network fails to provide a detailed and fully accurate reconstruction (c.f. the urban example in Fig. 6). It has to be stressed again, however, that the dataset used for training of the DSen2-CR model is globally sampled, which means that the network needs to learn a highly complex mapping from SAR to optical imagery for virtually every land cover type existing. By restricting the dataset or fine-tuning the model to a specific region or land cover type, it is expected that the SAR-to-optical translation results would improve significantly.

## 6 Summary and conclusion

In this paper, we have presented a deep residual neural network for cloud-removal in single-temporal Sentinel-2 satellite imagery. The main features of the proposed approach are threefold: On the one hand, we have incorporated a data fusion strategy to the cloud removal process in order to provide further information about the surface characteristics of the target scene based on Sentinel-1 SAR imagery. On the other hand, we have proposed a cloud-adaptive loss to circumvent the problem that cloud-affected and cloud-free training images can never be acquired at the same time. Finally, we have trained our model on a dataset sampled across the globe and over all meteorological seasons. Based on a deterministic split of training and test data, our experiments confirm the generic applicability of the final cloud-removal model. Both qualitative and quantitative results show that both the SAR-optical data fusion component and the cloud-adaptive training loss help significantly to predict reasonable cloud-free image content. In many cases, the pixel spectra are also improved. Due to the free availability of both Sentinel-2 and Sentinel-1 satellite imagery for all regions of the Earth, it is expected that the presented cloud-removal approach will be beneficial to a more temporally seamless monitoring of our environment.

## Declaration of Competing Interest

None.
